# Lumpy Skin Disease Virus with Four Knocked Out Genes Was Attenuated In Vivo and Protects Cattle from Infection

**DOI:** 10.3390/vaccines10101705

**Published:** 2022-10-12

**Authors:** Olga Chervyakova, Aisha Issabek, Kulyaisan Sultankulova, Arailym Bopi, Nurlan Kozhabergenov, Zamira Omarova, Ali Tulendibayev, Nurdos Aubakir, Mukhit Orynbayev

**Affiliations:** Research Institute for Biological Safety Problems, Gvardeiskiy 080409, Kazakhstan

**Keywords:** virus, lumpy skin disease, attenuation, homologous recombination, temporary dominant selection, safety, immunogenicity, protection, vaccine, vaccine vector

## Abstract

Vaccination with live attenuated vaccines is a key element in the prevention of lumpy skin disease. The mechanism of virus attenuation by long-term passaging in sensitive systems remains unclear. Targeted inactivation of virulence genes is the most promising way to obtain attenuated viruses. Four virulence genes in the genome of the lumpy skin disease virus (LSDV) Dermatitis nodulares/2016/Atyrau/KZ were sequentially knocked out by homologous recombination under conditions of temporary dominant selection. The recombinant LSDV Atyrau-5BJN(IL18) with a knockout of the LSDV005, LSDV008, LSDV066 and LSDV142 genes remained genetically stable for ten passages and efficiently replicated in cells of lamb testicles, saiga kidney and bovine kidney. In vivo experiments with cattle have shown that injection of the LSDV Atyrau-5BJN(IL18) at a high dose does not cause disease in animals or other deviations from the physiological norm. Immunization of cattle with the LSDV Atyrau-5BJN(IL18) induced the production of virus-neutralizing antibodies in titers of 4–5 log2. The challenge did not cause disease in immunized animals. The knockout of four virulence genes resulted in attenuation of the virulent LSDV without loss of immunogenicity. The recombinant LSDV Atyrau-5BJN(IL18) is safe for clinical use, immunogenic and protects animals from infection with the virulent LSDV.

## 1. Introduction

Lumpy skin disease virus (LSDV), a member of the genus *Capripoxvirus* of the *Poxviridae* family, causes an economically harmful disease in cattle in many regions of the world. The disease is characterized by an increase in body temperature, the formation of necrotic skin nodes (tubercles), generalized lymphadenitis, swelling of the extremities, damage to the eyes and mucous membranes of the respiratory, reproductive and digestive organs. The economic damage includes a sharp decrease in dairy products, the quality of milk and raw hides, together with loss of body weight, abortions and stillbirths, infertility and, in some cases, death of animals from opportunistic microflora; there are also substantial costs for treatment and veterinary and sanitary measures. In addition, for states focused on the export of livestock products, outbreaks of lumpy skin disease may lead to a ban on the export of cattle and slaughter products of this animal species [1].

Vaccination of animals with attenuated live vaccines is the main measure for the prevention and complete elimination of lumpy skin disease [2,3]. Attenuated capripoxviruses currently used for vaccination were obtained as a result of multiple passages in cell culture and/or chicken embryos [4]. In this case, attenuation is due to spontaneous mutations in the viral genome, affecting not only virulence genes, but also genes whose functions determine the replicative properties of the virus and the range of hosts sensitive to it [5]. Targeted removal/inactivation of virulence genes in the viral genome using genetic engineering methods is the most promising direction in the creation of highly attenuated poxviruses [6,7].

Based on a comparative analysis of poxvirus genomes, potential virulence genes have been identified for capripoxviruses [8,9]. Moreover, if the functions of most of the vaccinia virus genes have been experimentally confirmed, then the capripoxvirus genome still needs to be studied. Single studies on the function of capripoxvirus genes showed that the deletion of the ORF008-ORF018 and ORF066 (thymidine kinase) genes of the goat pox virus led to a significant attenuation of the virus and had no effect on replication activity [10]. Sheep pox virus was attenuated by deletion of the ORF019 gene, which encodes a kelch-like protein and is a virulence factor for capripoxviruses [11]. At the same time, single deletions of the LSD virus ORF005 or ORF008 genes retained the virulent phenotype of the virus [12].

It was also found that the removal of such genes as ORF020 (small subunit ribonucleotide reductase gene) [13], ORF066 (thymidine kinase gene) [14], ORF142 (secreted virulence factor) [15], and ORF011 (chemokine receptor) does not affect the replicative activity of the virus [16]. Cumulative removal of a number of virulence genes can, however, result in a stable attenuated immunogenic lumpy skin virus. Various approaches can be used to remove virulence genes [17].

In this study, we sequentially knocked out four virulence genes in the lumpy skin disease virus genome using homologous recombination under transient dominant selection conditions. The knockout of the LSDV005, LSDV008, LSDV142 genes was carried out by completely deleting the coding sequence; the reading frame was broken in the LSDV066 gene due to the insertion of a foreign sequence. Lumpy skin disease virus with four gene knockout as a potential vaccine candidate was evaluated for genetic stability, ability to replicate in sensitive cell lines, safety for clinical use, immunogenic activity and protectiveness in cattle.

## 2. Materials and Methods

### 2.1. Cells and Viruses

Cell cultures from the Cellular Biotechnology Laboratory of the Research Institute for Biological Safety Problems, Kazakhstan (RIBSP, RK) were used in this study. Primary lamb testicle (LT) cells were grown at 37 °C under 5% CO_2_ in semi-synthetic near-wall medium (SNM, RIBSP, RK) supplemented with 10% (*v*/*v*) fetal bovine serum (FBS, Sigma, St. Louis, MO, USA).

The virulent LSDV strain Dermatitis nodulares/2016/Atyrau/KZ (Atyrau/KZ), isolated during an outbreak in Kazakhstan in 2016 [18] and provided by the RIBSP Microbial Collection, was used as parental/wild-type virus to construct recombinant attenuated virus and to challenge vaccinated and control animals. LSDV Atyrau/KZ and its derivatives were propagated and subjected to titer determination in LT cells.

### 2.2. Construction of the Integration Plasmids

**Figure 1:**
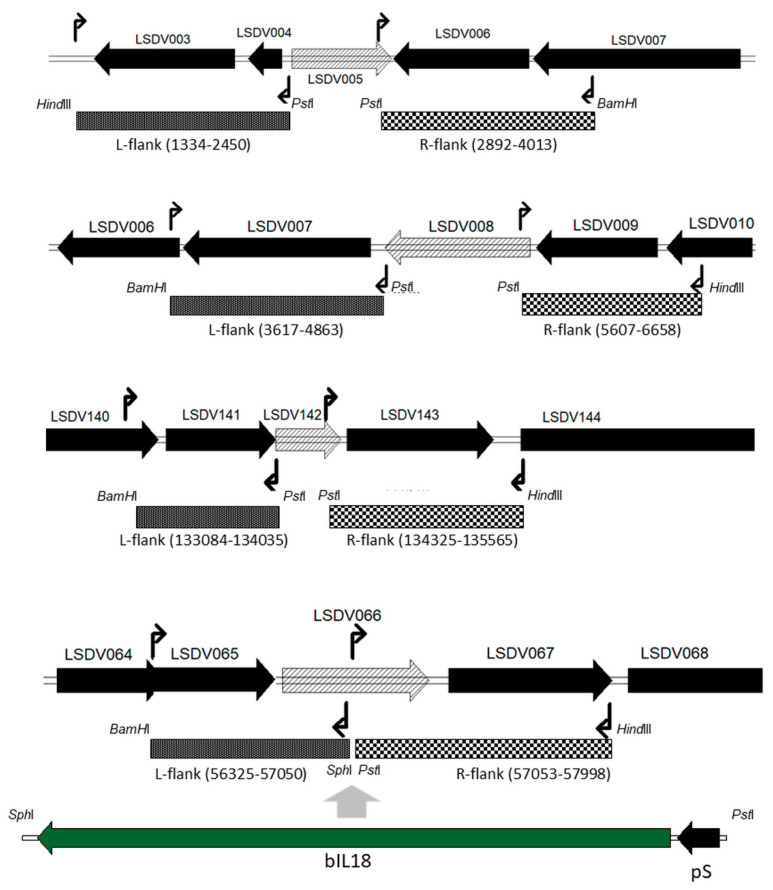
Scheme for obtaining PCR DNA fragments flanking the deletable LSDV genes for constructing integration plasmids. The black broken arrows indicate the locations of the oligonucleotide primers. The positions of the amplified DNA fragments in the viral genome are indicated for NCBI Reference Sequence: NC_003027.

Integration plasmids to remove the LSDV005, LSDV008 and LSDV142 genes from the LSDV genome were constructed based on plasmid pP7.5Kgpt. Plasmid pP7.5Kgpt contains the *E. coli* xanthine-guanine-phosphoribosyl-transferase gene sequence (*gpt*) under the control of the Camelpox virus 7.5K promoter [19]. Lumpy skin disease virus genome fragments (strain Atyrau/KZ) flanking the deletable gene on the left (L-flank) and on the right (R-flank) were obtained by PCR (Figure 1). Both DNA fragments flanking the deleted gene were inserted simultaneously into the pP7.5Kgpt plasmid at the BamHI and HindIII sites. Cleavage sites for restriction enzymes were introduced into the nucleotide sequence of the PCR primers (Figure 1).

The LSDV066 gene was knocked out as a result of a translation frame failure due to the insertion of a construct for the expression of foreign genes under the control of a synthetic vaccinia virus promoter [20]. The pIN-LSDV066-IL18 integration plasmid used in these studies provided for the insertion into the viral genome of a sequence encoding bovine interleukin-18 (IL18) mRNA.

The structures of plasmids pIN-LSDV005Δ, pIN-LSDV008Δ, pIN-LSDV142Δ and pIN-LSDV066-IL18 were confirmed by restriction analysis and sequencing.

### 2.3. Generation of Recombinant Lumpy Skin Disease Viruses with Knocked out Virulence Genes

Recombinant viruses were generated by homologous recombination under temporary dominant selection conditions as previously described [20]. Briefly, a monolayer of LT cells was infected with LSDV Atyrau/KZ or its derivatives at a dose of 0.1 PFU/cell for 2 h, and then transfected with one of the integration plasmids using Lipofectamine 2000 (Invitrogen, Carlsbad, CA, USA) according to the manufacturer’s protocol. When the cytopathic effect (CPE) reached 80% (three to five days), the cells were lysed with three freeze-thaw cycles. To enrich the transfection pool with recombinant LSDV, 2–3 consecutive passages were performed in a selective medium (SNM containing 2% FBS, 2.5 µg/mL mycophenolic acid, 25 µg/mL xanthine, and 1.5 µg/mL hypoxanthine). The obtained recombinant viruses were purified by the plaque method without selective pressure.

Recombinant viruses were identified by PCR. Targeted deletions/insertions were detected in viral genomes using appropriate primer pairs (Table 1). PCR analysis was performed at each selective passage also to determine the homogeneity of the recombinant virus, i.e., total absence of parental virus.

To identify recombinant viruses, PCR was performed in 25 µL containing 5 µL 5× OneTaq Standard reaction buffer (NEB, Ipswich, MA, USA), 0.5 µL 10 mM dNTPs (NEB, Ipswich, MA, USA), 1 µL of each primer (10 pmol/µL), and 0.125 µL One Taq DNA polymerase (0.625 Units, NEB, Ipswich, MA, USA) 1 µL template DNA (100 ng/µL), and sterile distilled water to the final reaction volume. Template DNA was denatured for 30 s at 94 °C, primer annealing was carried out at 50 °C for 30 s, and strand extension was at 72 °C for 90 s (repeated through 30 amplification cycles).

### 2.4. Western Blotting

Western blots were performed as previously described using polyclonal mouse anti-bIL18 sera [19].

### 2.5. Animals

In the research, cattle obtained from farms free from lumpy skin disease were used. Animals were brought to the experimental animal’s wing of the Research Institute for Biological Safety Problem. Blood sera of all animals were examined in the virus neutralization test for the absence of antibodies to lumpy skin disease virus and other capripoxviruses. All experimental procedures and animal care were performed in compliance with institutional animal care regulations. The experiment protocol was approved by the Committee on the Ethics of Animal Experiments of the Research Institute for Biological Safety Problems RK (Permit Number 06/2021, approved 20 August 2021).

### 2.6. Safety of LSDV Atyrau-5BJN(IL18) in Cattle

To assess the safety of LSDV Atyrau-5BJN(IL18), three calves up to six months of age and three bulls of two years of age were subcutaneously injected into the middle third of the neck with LSDV Atyrau-5BJN(IL18) at a dose of 100,000 TCID50/2 mL. Animals of the control group (three calves under the age of six months) were injected with an equal volume of PBS. The animals were observed for 21 days; the general clinical condition, body temperature, presence and size of pockmarks were assessed.

### 2.7. Immunogenicity and Protectiveness of LSDV Atyrau-5BJN(IL18) in Cattle

Immunogenicity was assessed by the ability of the virus Atyrau-5BJN(IL18) to induce the production of virus-neutralizing antibodies. In the experiment, eight clinically healthy cattle aged 6–8 months were used, in the blood sera of which there were no virus-neutralizing antibodies to capripoxviruses. Animals were randomly divided into two groups. Animals of the 1st group (n = 6) were injected subcutaneously with LSDV Atyrau-5BJN(IL18) at a dose of 6000 TCID50/mL. Animals of the 2nd group (n = 2) were injected with PBS (control).

All animals were kept separately in isolated rooms. Every day for 21 days after vaccination, a clinical examination of animals was performed with the measurement of rectal temperature. Serum samples were collected at 0, 7, 14 and 21 days after vaccination and serum-neutralizing antibody titers to LSDV Atyrau/KZ were determined by plaque reduction on LT cell monolayers [21].

On the 21st day after vaccination, the animals of the first and second groups were challenged with a virulent strain of LSDV Atyrau/KZ by subcutaneous inoculation in the middle third of the neck at a dose of 100,000 TCID50/1 mL. The infected animals were clinically observed for 21 days with daily body temperature measurements. The number of clinically healthy challenged cattle assessed the protective efficacy.

### 2.8. Statistical Analyses

Antibody responses are expressed as the mean ± standard deviation of the mean of three independent experiments. *p* < 0.05 were considered significant. Statistical analysis of experimental data was performed by using Graphpad Prism, version 6.0, Graphpad Software Inc. (San Diego, CA, USA).

## 3. Results

### 3.1. Generation of Recombinant Viruses with Targeted Knock out of Virulence Genes

Sequential removal of the target virulence genes was performed by homologous recombination using the constructed integration plasmids pIN-LSDV005Δ, pIN-LSDV008Δ, pIN-LSDV066-IL18 and pIN-LSDV142Δ. For the selection of recombinants, the method of temporary dominant selection was used, which made it possible to sequentially remove a number of genes using the same selective marker (*gpt*). Under the conditions of *gpt*-selection, plasmid DNA was completely integrated into the viral genome with the formation of an unstable genetic construct. After removal of selective pressure and intramolecular recombination of the viral genome, two types of viruses are formed: the original and recombinant with the deleted target gene. Intramolecular recombination also led to the removal of the *gpt* gene from the viral genome [20,22].

**Figure 2:**
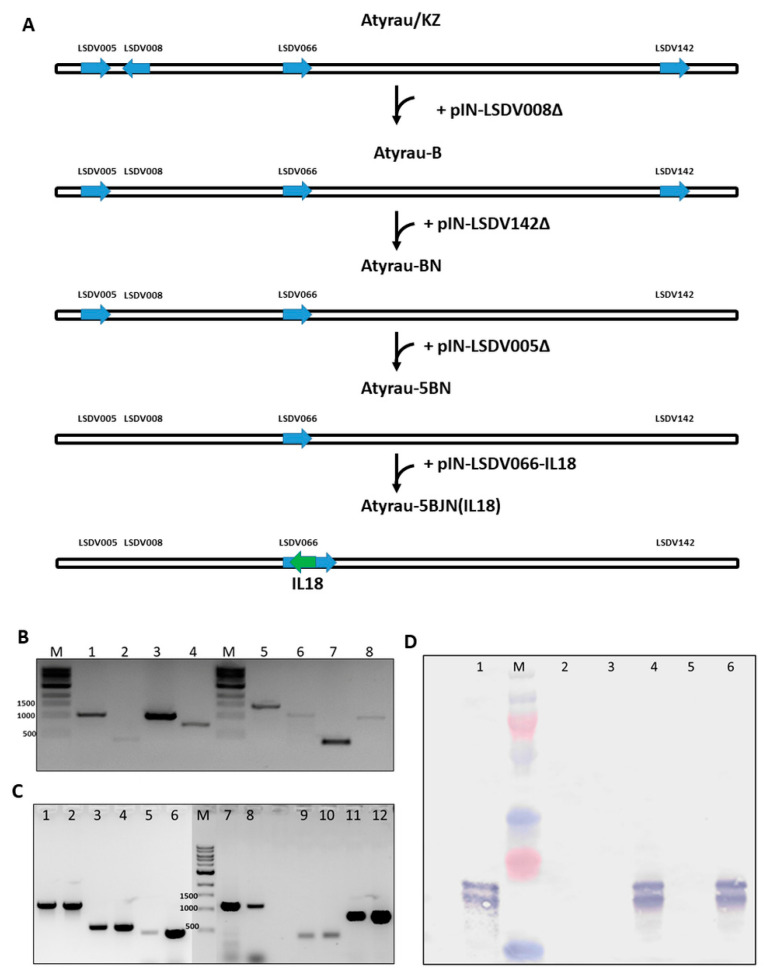
Construction of recombinant LSDV Atyrau-5BJN(IL-18) and its analysis. (**A**) Scheme for obtaining recombinant LSDV with a knockout of four virulence genes. The sequence of introducing mutations into the viral genome is shown. (**B**) Analysis of the presence of target deletions/insertions: M—DNA Ladder 1 kb, N3232, NEB; 1–4—recombinant LSDV Atyrau-5BJN(IL18); 5–8—parental LSDV Atyrau/KZ; 1,5—LSDV005 locus; 2,6—LSDV008 locus; 3,7—LSDV066 locus; 4,8—LSDV142 locus. (**C**) Analysis of the genetic stability of the virus after the tenth passage: M—DNA Ladder 1 kb, N3232, NEB; 1,2—LSDV066 locus; 3,4—interleukin 18; 5,6—*gpt* (plasmid DNA); 7,8—LSDV005 locus; 9,10—LSDV008 locus; 11,12—LSDV142 locus; 1,3,5,7,9,11—DNA of the recombinant virus; 2,4,6,8,10,12—corresponding integration plasmids (positive control). (**D**) Immunodetection of interleukin 18 expressed by recombinant lumpy skin disease virus in LT cell culture: M—protein molecular weight marker; 1—bacterially expressed interleukin 18 (positive control); 2,5—lysate of uninfected cells (negative control); 3—lysate of cells infected with parental LSDV Atyrau/KZ; 4—lysate of cells infected with recombinant LSDV Atyrau-5BJN(IL18), passage 1; 6—lysate of cells infected with recombinant LSDV Atyrau-5BJN(IL18), passage 10.

At the first stage, a monolayer of LT cells was infected with the virulent virus Atyrau/KZ and transfected with pIN-LSDV008Δ plasmid DNA under *gpt*-selection conditions. Recombinant viruses Atyrau-B with a deletion of the LSDV008 gene were detected by PCR analysis (see Supplementary Data). One of the recombinant clones Atyrau-B was used to remove the LSDV142 gene. As a result, the Atyrau-BN recombinant was obtained with the deletion of two genes LSDV008 and LSDV142. Next, the LSDV005 and LSDV066 genes were disrupted (Figure 2A). When the LSDV066 gene was disrupted, the sequence encoding bovine interleukin-18 mRNA was integrated into the viral genome. Deletions and insertions were detected by PCR (see Supplementary Data). As a result, a recombinant LSDV Atyrau-5BJN(IL18) was obtained with a knockout of four virulence genes. PCR analysis showed that the amplification products of the target genes LSDV005, LSDV008, LSDV066, and LSDV142 of the recombinant LSDV Atyrau-5BJN(IL18) were 1152, 432, 1085, and 816 bp, respectively, while the size of the parent LSDV Atyrau/KZ was 1587, 1169, 413 and 1102 bp, respectively, which was consistent with the calculated values (Table 1, Figure 2B).

Recombinant LSDV Atyrau-5BJN(IL-18) replicated efficiently in LT, MDBK (Madin-Darby bovine kidney line) and SK (saiga kidney cell line) cells during at least ten passages. The maximum activity of the virus (5.5–6.0 lgTCID50/mL) was noted on days 4–5 of cultivation in LT and MDBK cells. Virus titer in SK cells was 5.25–5.5 lg TCID 50/mL. The reproduction curves of the recombinant and the original virus did not differ statistically significantly from each other.

It was found that recombinant LSDV Atyrau-5BJN(IL18) with a knockout of four genes remained genetically stable for 10 passages (Figure 2C). Expression of the inserted interleukin 18 gene was confirmed by Western blot (Figure 2D).

### 3.2. Safety of Recombinant LSDV Atyrau-5BJN(IL18)

**Figure 3:**
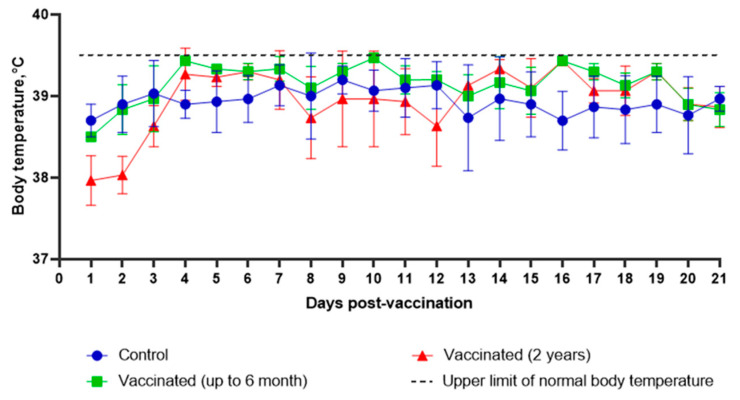
Changes in body temperature of cattle injected with 100,000 TCID50 of LSDV Atyrau-5BJN(IL18).

The safety of LSDV Atyrau-5BJN(IL18) in cattle has been studied. The animals were clinically observed for 21 days. During the entire observation period, all vaccinated animals remained clinically healthy. An increase in body temperature was not detected (Figure 3). At the same time, on the 5th day after the introduction of the virus, small swelling up to 2 cm in size were detected in two calves at the injection site, which resolved on the 11–15th day of observation. On day 21, all animals remained clinically healthy and no deviations from the physiological norm were detected. Thus, recombinant LSDV Atyrau-5BJN(IL18) is safe for cattle.

### 3.3. Immunogenicity and Protectiveness of LSDV Atyrau-5BJN(IL18)

**Figure 4:**
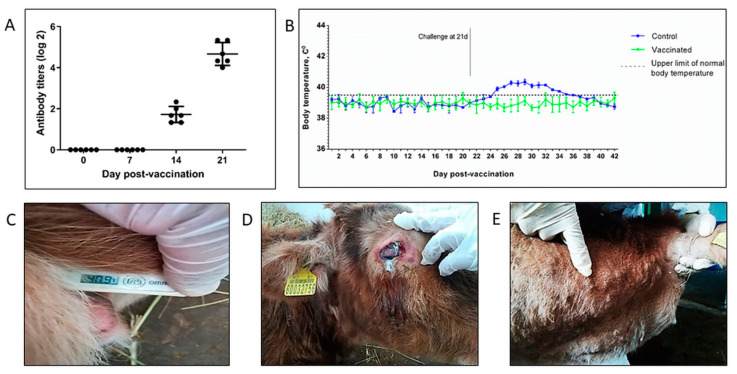
Immunogenicity and protectiveness of recombinant LSDV Atyrau-5BJN(IL18) in cattle. (**A**) Virus neutralizing antibody titers after immunization of animals. The circles indicate the values of the titer of virus-neutralizing antibodies for each animal. The lines show the mean and standard deviation. (**B**) The changes in body temperature of the cattle after vaccination or challenge. (**C**–**E**) Clinical signs of infection in control animals after challenge.

The immunogenicity of LSDV Atyrau-5BJN(IL18) was assessed by the level of virus-neutralizing antibodies in the blood serum of cattle obtained 7, 14, and 28 days after immunization. Figure 4A shows that recombinant LSDV Atyrau-5BJN(IL18) elicits neutralizing antibodies in subcutaneously immunized animals. On day 14 after immunization, the titer of virus-neutralizing antibodies was 1.72 ± 0.39 log2, and by day 21, it reached 4.67 ± 0.56 log2.

Vaccinated and control animals were challenged with the virulent LSDV Atyrau/KZ at 21 days after vaccination. An increase in temperature to 40.9 °C (Figure 4C) was noted in the animals of the control group on the fourth day, which was accompanied by lacrimation and serous-mucous discharge from the nose (Figure 4D). On the seventh day, dense rounded nodules that were easily palpable formed on the skin of the animals (Figure 4E). On days 10–14, the epidermis separated along the edges of the nodules, a depression formed in the center, and tissue necrosis began. The vaccinated animals remained clinically healthy; no increase in body temperature was recorded.

Thus, LSDV Atyrau-5BJN(IL18) demonstrated satisfactory immunogenicity and was able to protect cattle from infection with the virulent LSDV Atyrau/KZ.

## 4. Discussion

The outbreak of lumpy skin disease in Kazakhstan in 2016 and the worsening epizootic situation of lumpy skin disease in the countries bordering Kazakhstan prompted research to develop effective attenuated vaccines [18]. According to WOAH recommendations, live attenuated strains of capripoxviruses should be used to prevent lumpy skin disease [23]. Capripoxviruses (sheep pox virus, goat pox virus and lumpy skin disease virus) have high genetic homology [8,24] and, theoretically, one attenuated strain can be used to protect against infections [25]. The experience of combating the disease shows that only the use of vaccines from homologous viruses leads to its elimination [3]. The Veterinary Service of Kazakhstan also recommends the use of attenuated homologous virus vaccines. Therefore, the virulent LSDV Atyrau/KZ was taken as the basis for the development of a live attenuated vaccine. Various approaches are used to attenuate viruses. The classic method of attenuation is multiple passaging of viruses in permissive and non-permissive cell cultures and/or chick embryos [26]. Many point mutations accumulate in the viral genome during of passages. As a result, the virulence of viruses is reduced or lost. At the same time, the molecular basis of virus attenuation remains unidentified and there is a risk of reversion to the virulent type. An alternative to the classical method of virus attenuation is the introduction of targeted mutations into the viral genome. Currently, a large number of poxvirus genes encoding virulence factors have been identified. “Switching off” (knockout) of these genes leads to the attenuation of viruses. Knockout of genes can be carried out via their complete or partial removal or via disruption of the reading frame by inserting a foreign sequence [27].

In this study, four genes encoding molecular virulence factors were knocked out to attenuate the virulent lumpy skin disease virus Atyrau/KZ. Based on the analysis of published data, we selected the LSDV005, LSDV008, LSDV066 and LSDV142 virus genes [8,9].

The LSDV005 gene encodes an interleukin-10-like protein, also identified in poxviruses such as Orf [28] and Yaba-like disease virus [29]. Interleukin-10 is a multifunctional cytokine with immunostimulatory and immunosuppressive effects [30]. The poxvirus interleukin-10-like protein promotes immune response evasion by mimicking the suppressive effect of host interleukin-10 on Th1-mediated responses [31]. The deletion of this gene did not affect the coding regions of the LSDV004 and LSDV006 genes and reduced the length of the viral genome by 435 bp.

The second deleted gene was LSDV008, which is a homologue of the vaccinia virus B8R gene and encodes an interferon-γ receptor-like protein. Poxvirus interferon-γ receptor like proteins usually show limited similarity to the extracellular domains of mammalian interferon-γ receptor and probably competitively prevent interferons from binding to their native receptors [32]. The role of poxvirus interferon-γ receptor like protein has been demonstrated in studies by Nash et al. [33]. Deletion of the LSDV008 gene reduced the viral genome by another 737 bp without affecting neighboring ORFs.

Another deleted gene was LSDV142, a homologue of the N1L gene of the vaccinia virus, which encodes a protein of the Bcl-2-like protein family and inhibits both apoptosis and activation of the pro-inflammatory transcription factor NF-κB (nuclear factor kappa B) [34,35]. Thus, deletion of the N1L (GTPV135) gene in the goat poxvirus genome led to lesser attenuation of the virus than the deletion of the thymidine kinase gene. However, goatpox virus expressing the PPRV H gene inserted at the GTPV135 locus elicited significantly higher neutralizing antibody responses to PPR and GTPV than those obtained using the tk locus as the insertion site [15]. Deletion of the LSDV142 gene reduced the Atyrau/KZ virus genome by another 286 bp.

Knockout of the LSDV066 gene encoding thymidine kinase occurred as a result of translation frame failure due to the insertion of a foreign sequence. A sequence encoding bovine interleukin-18 mRNA under the control of a synthetic early-late vaccinia virus promoter was inserted into the thymidine kinase locus. Recombinant LSDV Atyrau-5BJN(IL18) stably expressed interleukin 18 in vitro in LT cells.

The presence of deletions and insertions was confirmed by both PCR and viral genome sequencing (GenBank ID: ON005067) [36].

It was found that the recombinant LSDV Atyrau-5BJN(IL18) retained its genetic stability for ten passages and replicated efficiently in LT, MDBK and SK cells. Knockout of four genes did not affect virus replication in vitro. In vivo experiments with cattle showed that the recombinant virus did not cause clinical signs of infection when administered at high doses of the virus, which indicates its safety for clinical use. Immunization of animals with the recombinant LSDV Atyrau-5BJN(IL18) at a dose of 6000 TCID50 induced the production of virus-neutralizing antibodies and protected the animals from infection with the virulent LSDV. In contrast, control animals that received saline developed lumpy skin disease after challenge with a virulent virus.

Thus, LSDV Atyrau-5BJN(IL18) demonstrated high safety and immunogenicity and has the potential to be used as a new vaccine for the prevention of lumpy skin disease in cattle. The ability to stably express foreign genes (in this case interleukin-18) demonstrates the potential of using this virus also as a vaccine vector.

## 5. Patents

An application for the invention “Attenuated Lumpy skin disease virus, strain Atyrau-5BJN(IL18) for the preparation of specific prophylaxis means” was submitted to the National Institute of Intellectual Property of the Republic of Kazakhstan No. 2022/0568.1 dated 21 September 2022.

## Figures and Tables

**Table 1:** Primer utilized for detecting deletions/insertions in the LSDV genomes by PCR.

Target Gene	Primer Name	Sequence (5′–3′)	Product Size, bp	
			Recombinant Type	Wild Type
LSDV005	RCR005ΔF	agtagtatttaccaccaacatg	1152	1587
	RCR005ΔR	caagtatgatgatataataacg		
LSDV008	RCR008ΔF	tcaaatactttagactcttccc	432	1169
	RCR008ΔR	agggtaagtatccaagtttt		
LSDV066	RCR-TK-F	aattataggacctatgttttctggc	1085	413
	RCR-TK-R	cagcgtctttataacattccat		
LSDV142	RCR142ΔF	tataagatgtcgattcccag	816	1102
	RCR142ΔR	attttgtgacttgtgcgc		

## Data Availability

The genome sequence of the lumpy skin disease virus Atyrau-5BJN(IL-18) has been deposited in GenBank under accession number ON005067 (https://www.ncbi.nlm.nih.gov/nuccore/ON005067 (accessed on 3 October 2022)), and the raw data have been submitted to the SRA under BioProject number PRJNA825391 (https://www.ncbi.nlm.nih.gov/sra/?term=PRJNA825391 (accessed on 3 October 2022)).

## Supplementary Materials

The following supporting information can be downloaded at: https://www.mdpi.com/article/10.3390/vaccines10101705/s1, Supplementary File: The results of PCR analysis of intermediate recombinant viruses in the process of constructing lumpy skin disease virus with a knockout of four genes.
