# Supplementary material for: Lumpy Skin Disease Virus with Four Knocked Out Genes Was Attenuated In Vivo and Protects Cattle from Infection
Source: Vaccines (Basel). 2022 Oct 12;10(10):1705. doi: 10.3390/vaccines10101705 (PMC9610274; doi:10.3390/vaccines10101705)
Supplement: Supplementary file 1 [file vaccines-10-01705-s001.zip › vaccines-1945636-supplementary.pdf]

1. Obtaining a recombinant lumpy skin disease virus with a knockout of the LSDV008 gene

As a result of the first cloning, 48 clones were obtained, of which, according to the results of PCR analysis (Figure S1), clone 1B2 was selected for further work.

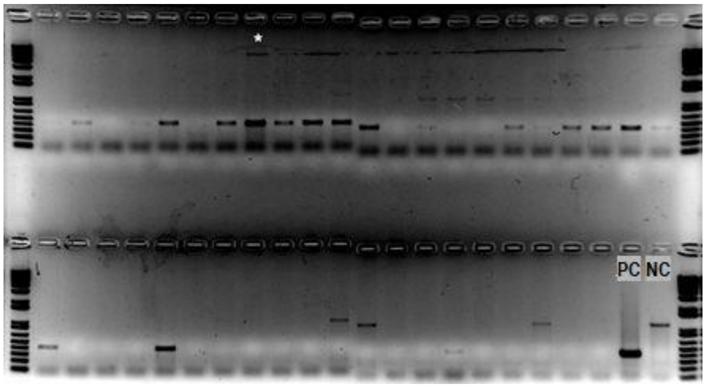

PC - positive control, NC - negative control, (*) the chosen clone is marked with an asterisk

Figure S1 - PCR analysis of viral clones LSDV008Δ

Viral clone 1B2 was further cloned. The resulting clones were analyzed for the structure of the locus and the presence of plasmid DNA in the viral genome. It was found that almost all obtained clones have the recombinant genotype of the locus (Figure S2A), however, some of the clones had plasmid DNA in the genome (Figure S2B).

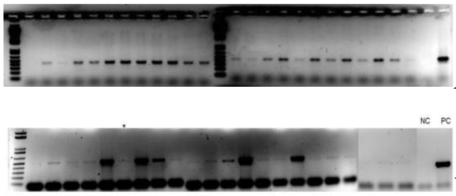

A

B

A - analysis of the structure of the 008 locus, B - analysis for the presence of plasmid DNA in the viral genome, NC - negative control, PC - positive control, (*) the selected clone is marked with an asterisk (7A6)

Figure S2 - Results of PCR analysis of clones of the 1B2 LSDV008Δ virus obtained by the method of limiting dilution

Viral clone 7A6 was cloned by the plaque method. As a result of the analysis of the obtained clones of the virus, two clones 42A6 and 42C6 (marked with an asterisk) were selected, which had the recombinant type of the locus 008 (Figure S3A) and there was no plasmid DNA in the viral genome (Figure S3B).

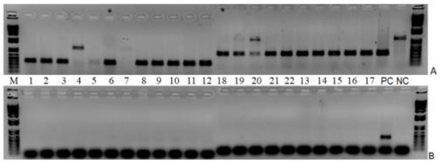

A - analysis of the structure of the 008 locus, B - analysis for the presence of plasmid DNA in the viral genome, NC - negative control, PC - positive control, (*) selected clones (42A6 and 42C6) are marked with an asterisk

Figure S3 - Results of PCR analysis of clones of the 7A6 LSDV008Δ virus obtained by the agarose-coated plaque method

The determination of the stability of the obtained recombinants was carried out by five consecutive passages in cell culture. At each passage level, the structure of the locus was determined. As a result of the studies, it was found that during five passages the recombinant virus remains genetically stable at the 008Δ locus (Figure S4).

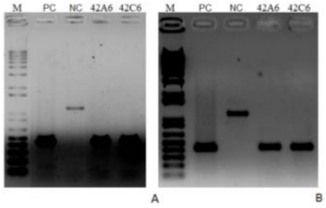

A - first passage, B - fifth passage, NC - negative control, PC - positive control

Figure S4 - PCR analysis of genetic stability of recombinant viruses LSDV008Δ

Thus, the recombinant virus with the deletion of the LSDV008 gene retains the recombinant genotype for five passages (observation period). The deletion of this gene does not affect the replication activity of the virus in the LT cell culture.

2 Obtaining a recombinant lumpy skin disease virus with a knockout of two genes LSDV008 and LSDV142

To obtain a virus with a knockout of two genes, we used the recombinant LSDV008Δ clone 7A6 obtained by us. The cell culture infected with LSDV008Δ/7A6 virus was transfected with pIN-LSDV142Δ plasmid DNA. The cell lysate after transfection was passaged under selective conditions and then cloned by the plaque method under agarose coating. Twenty-two single viral plaques were selected and expanded. The resulting viral clones were analyzed by PCR for the structure of the loci. It was found that only three clones had a deletion of the LSDV142 gene (Figure S5A, clones are indicated by asterisks). At the same time, one of them was a mixture of viruses of recombinant and original types. Analysis of clones for the presence of a deletion of the LSDV008 gene showed that not all clones had it (Figure S5B). This is due to the inhomogeneity of the 7A6 viral clone used for recombination.

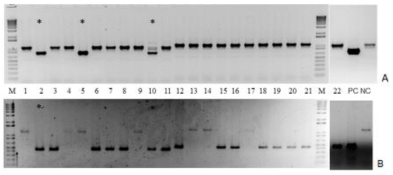

A – analysis of the structure of locus 142, B – analysis of the structure of locus 008, NC – negative control, PC – positive control, (*) selected clones (46A2, 46A5, 46B4)

Figure S5 - Results of PCR analysis of virus clones with a deletion of two genes obtained by the agarose-coated plaque method

Viral clones with two deletions 46A2 and 46B4 (marked with asterisks in Figure S5B) were tested for the presence of plasmid DNA in the viral genome (Figure S6). It was found that only clone 46A2 did not have plasmid DNA in the structure of the viral genome. The genetic stability of clone 46A2 was confirmed by PCR after five passages in cell culture.

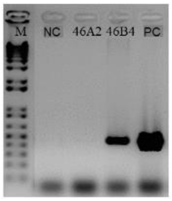

NC - negative control, PC - positive control

Figure S6 - Analysis of viral clones with a deletion of two genes for the presence of plasmid DNA in the viral genome

Thus, a lumpy skin disease virus with a knockout of two genes was obtained. The recombinant virus is conventionally designated LSDV-BN.

3 Production of recombinant lumpy skin disease virus with knockout of three genes

To obtain a virus with a knockout of three genes, we used the recombinant LSDV-BN obtained by us. The cell culture infected with LSDV-BN virus was transfected with plasmid DNA pIN-LSDV005Δ. Cell lysates after transfection were passaged under selective conditions and then cloned by the plaque method under agarose coating. As a result of cloning, 23 plaques were selected, which were individually propagated and analyzed by PCR for the structure of the locus 005. As a result, it was found that only two clones had a mixed genotype: 51A1 and 51D4 (Figure S7, clones are marked with an asterisk).

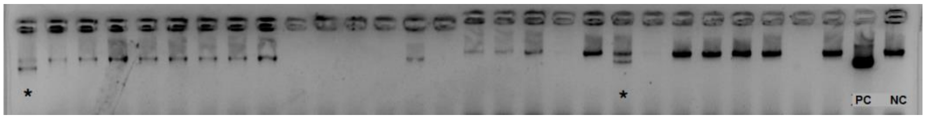

* - clones with a mixed genotype, PC - positive control (recombinant genotype), NC - negative control (parental genotype)

Figure S7 - Analysis of the structure of the 005 locus of viral clones (plate 51)

Clones with a mixed genotype were serially cloned by the method of limiting dilution and plaques. As a result of the second round of cloning, two clones 56C5 and 56D2 were isolated with a deletion of the 005 gene (Figure S8).

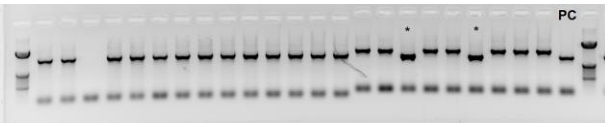

Figure S8 - Analysis of the structure of the 005 locus of viral clones

Thus, a lumpy skin disease virus with a knockout of three genes was obtained. The recombinant virus is conventionally designated LSDV-5BN.

4 Obtaining a recombinant lumpy skin disease virus with a four-gene knockout

The knockout of the quadruple gene in the viral genome was carried out by the method of insertional mutagenesis. The foreign sequence encoding the cDNA of bovine interleukin 18 was inserted into the thymidine kinase (LSDV066) gene of the recombinant LSDV-5BN virus (clone 56C5). The cell culture infected with LSDV-5BN virus was transfected with plasmid DNA pIN LSDV066-IL18. Cell lysates after transfection were passaged under selective conditions and then cloned by the plaque method under agarose coating. As a result of several rounds of cloning, 10 clones of the virus were obtained, encoding the cDNA of bovine interleukin 18 (Figure S9A, marked with an asterisk). Analysis of the locus structure showed that only one clone 68E7 (Fig. S9B, marked with an asterisk) has a locus structure characteristic of a stable recombinant.

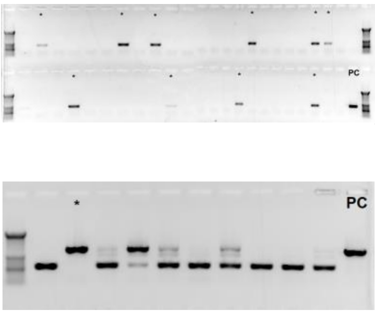

A

B

A - analysis for the presence of the inserted gene, B - analysis of the structure of the 066 locus, PC - positive control, (*) selected viral clones are marked with an asterisk

Figure S9 - Results of PCR analysis of virus clones with knockout of the fourth gene
